# Persistence of protective anti-poliovirus antibody levels in 4-year-old children previously primed with Picovax®, a trivalent, aluminium-adjuvanted reduced dose inactivated polio vaccine

**DOI:** 10.1016/j.vaccine.2022.06.084

**Published:** 2022-09-22

**Authors:** Xavier Sáez-Llorens, Milagros Chan, Rodrigo DeAntonio, Torben Petersen, Charlotte Olesen, Jens Søndergaard Jensen, Charlotte Sørensen, Lena Messerschmidt Ekstrand, Michaela Katrine Czort, Hans-Henrik Kristensen, Niels Thulstrup, Dorte Birk Christoffersen

**Affiliations:** a Hospital del Niño, Panama City, Panama; b Cevaxin, Avenida Mexico Calle 33, Local 4, Panama City, Panama; c Sistema Nacional de Investigación, Senacyt, Panama; d Larix A/S, Lyskær 8b, 2730 Herlev, Denmark; e AJ Vaccines A/S, Artillerivej 5, 2300 Copenhagen S, Denmark

**Keywords:** Polio, Affordable inactivated polio vaccine, Oral polio vaccine, Immunogenicity, Aluminium hydroxide adjuvant, Reduced dose

## Abstract

**Background:**

To meet the demand for effective and affordable inactivated polio vaccines (IPVs), a reduced dose, aluminium hydroxide (Al(OH)_3_)-adjuvanted IPV vaccine was developed (IPV-Al, Picovax®) and evaluated in clinical trials. The present trial is an extension of two previous trials (a primary and a booster trial). The aim was to evaluate the persistence of seroprotective antibodies (poliovirus type-specific antibody titre ≥ 8) in 4-year-old children who previously received IPV–Al as primary and booster vaccine doses and to determine the potential booster response and safety profile of an additional dose of IPV-Al.

**Methods:**

Children participating in the two previous trials were invited to receive one additional dose of IPV-Al at 4 years of age (2.5 years after the booster dose) and to have their blood samples collected to measure the pre- and post-vaccination antibody titres. Systemic adverse events (AEs) and local reactogenicity were recorded.

**Results:**

At study entry, the seroprotection rates were 89.2%, 100% and 91.1% against poliovirus type 1, 2 and 3, respectively. The additional vaccination with IPV-Al boosted the level of poliovirus type 1, 2 and 3 antibodies to above the seroprotection threshold for all but one subject, i.e., 99.4% for type 1 and 100% for types 2 and 3. The additional dose induced a robust booster response of a 26.3-, 13.9- and 30.9-fold increase in titre for poliovirus types 1, 2 and 3, respectively. The vaccine was well tolerated, with only mild and transient AEs reported.

**Conclusions:**

The present trial demonstrated that the primary vaccination with an aluminium-adjuvanted reduced dose IPV induced a persistent immune memory as evidenced by the robust anamnestic response when the subjects were re-exposed to the antigen 2.5 years after the last dose. Thus, the IPV-Al is an efficient and safe addition to increase the availability of inactivated polio vaccines globally. (ClinicalTrials.gov reg no. NCT04448132).

## 1 Introduction

Vaccines against polioviruses have been instrumental in reducing the total number of cases of polio globally. In 1988, WHO started the Global Polio Eradication Initiative to eradicate polio globally and to stimulate the development of efficient, safe, and affordable vaccines. Since then, more than 2.5 billion children have been vaccinated, and the total cases of polio fell from 350,000/year to 22 reported cases in 2017 worldwide [1]. Of the three distinct serotypes of the wild poliovirus (WPV), types 2 and 3 were declared eradicated in 2015 and 2019, respectively. A recent rise in WPV type 1 cases up to 140 in 2020, seen in Pakistan and Afghanistan, highlights the importance of a continuous vaccination campaign, particularly in high-risk and endemic areas, low resource countries or armed conflict zones [1].

The trivalent oral polio vaccine (OPV) has accelerated the eradication initiative because of its easy distribution, low cost, and oral applicability. However, due to low immunisation rates within certain communities, another form of poliovirus, namely, circulating vaccine-derived poliovirus (cVDPV), have been observed, with 959 cases occurring globally in 2020 [2]. This constitutes an inherent risk for children to develop vaccine-associated paralytic polio. This low but constant risk prompted the WHO to promote the shift first from trivalent OPV to bivalent (types 1 and 3) OPV, and later to inactivated polio vaccines (IPVs), which have no risk of inducing cVDPV [3]. However, the complete transition to IPV faces some challenges, including the limited availability of affordable IPV. AJ Vaccines A/S has developed an aluminium hydroxide (Al(OH)_3_)-adjuvanted IPV (IPV-Al), containing one-tenth of the standard dose of IPV, making it less costly. The use of Al(OH)_3_ as adjuvant for vaccines dates back more than a century and, for certain antigens, is an efficient way to stimulate an immune response [4]. A safe and efficient reduced dose, aluminium hydroxide adjuvanted IPV would be a significant contribution to increase the number of available and affordable IPV doses, and thereby help to meet the increasing global demand for IPV.

Previous clinical trials have demonstrated the non-inferiority of IPV-Al when compared with standard IPV. In a phase 2 trial in the Dominican Republic, Rivera and colleagues [5] found similar immunogenicity and safety profiles for three aluminium hydroxide adjuvanted reduced doses, i.e., 1/3, 1/5, and 1/10 dose, when compared with the standard IPV dose. The lowest dose of the vaccine (1/10) performed well and was selected for further clinical studies. Similar results were observed by Bravo and colleagues in a phase 3 trial in the Philippines using the 1/10 dose [6]. Another phase 3, observer-blinded, randomised, and multicentre primary vaccination trial was conducted in Panama with infants vaccinated with three doses of either IPV–Al or standard IPV at 2, 4 and 6 months of age. This was followed by an extension trial in which the same subjects all received a booster vaccination with IPV-Al at the age of 15–18 months. The post-booster seroprotection rates were 97% for poliovirus type 1 and 100% for type 2 and 3 [7]. These findings confirmed the feasibility of using Al(OH)_3_ as adjuvant to reduce the antigen content of IPV.

The present trial is a phase 4 extension trial conducted in Panama in 4-year-old subjects who received IPV-Al (Picovax®) in the primary and booster trials [7]. The aim was to determine the persistence of antibody titres of poliovirus type 1, 2 and 3 in these subjects, 2.5 years after receiving the booster dose of IPV-Al, as well as to measure their antibody responses to an additional dose of IPV-Al and to describe the safety profile of IPV-Al.

## 2 Methods

### 2.1 Trial design and subjects

This was a phase 4, multicentre, open-label clinical trial (ClinicalTrials.gov registration no. NCT04448132). The trial took place at four investigational sites in Panama. The trial protocol was approved by the relevant ethics committee. The trial was conducted according to the principles of good clinical practice [8] and the Declaration of Helsinki [9].

The subjects were 4 years of age, healthy, and were previously vaccinated with IPV-Al at 2, 4, 6 and 15–18 months of age; parents/legal guardians provided written informed consent and were granted access to their child’s medical and vaccination records. Key exclusion criteria were any previous vaccination with OPV or IPV outside the primary and booster trials, vaccination against yellow fever during and four weeks prior to the first trial visit, any known or suspected immunodeficiency, severe uncontrolled chronic disease and coagulopathy, known or suspected allergy to the vaccine constituents and any treatment with immune response modifiers and antipyretics/analgesics received/scheduled on the day of the vaccination(s) and the following 2 days. Some subjects from the preceding trials (primary/booster) could not either be reached or meet the inclusion criteria; thus, 167 subjects were screened for eligibility, corresponding to about 48% of the eligible candidates from the primary and booster trials.

### 2.2 Trial vaccinations

The investigational vaccine was IPV-Al (Picovax®, AJ Vaccines A/S), which per dose contains inactivated poliovirus type 1 (≥3.2 D-antigen units (DU)), type 2 (≥0.88 DU) and type 3 (≥3.1 DU), along with 2-phenoxyethanol (0.5%), and Al(OH)_3_ (0.5 mg), appearing as an opaque suspension for injection. A dose of 0.5 mL was administered intramuscularly in the right shoulder (deltoid muscle). Other routine childhood vaccines were administered concomitantly at different injection sites (Varicella vaccine in the left deltoid muscle and Diphtheria toxoid vaccine; Pertussis vaccine; Tetanus toxoid vaccine, DTwP/TdaP; or Diphtheria toxoid vaccine; Haemophilus influenzae type b vaccine; Pertussis vaccine; Tetanus toxoid vaccine, DTP-Hib, in the left anterolateral thigh).

### 2.3 Trial procedures

During visit 1, a baseline blood sample was taken, and the trial vaccine (IPV–Al) was administered. A diary, thermometer and ruler were handed out to the parent(s)/guardian(s) for measurements and recordings of injection site reactions (ISRs), axillary temperature and solicited adverse events (AEs). The parent(s)/guardian(s) were asked to record any occurrences of solicited AEs during the first 6 days after the vaccination. At visit 2, 28–42 days later, a blood sample was taken, concomitant medication was documented and AEs and ISRs recorded.

Blood samples were analysed using a validated Vero cell assay for the antibody measurements, which has been previously described [10].

### 2.4 Endpoints

The primary endpoint was the rate of seroprotected subjects against each poliovirus type, 28–42 days after administration of the additional IPV-Al dose (at visit 2). Seroprotection was defined as a poliovirus type-specific antibody titre ≥ 8 for the types 1, 2 and 3 [11], [12], [13]. Secondary immunogenicity endpoints included the proportion of seroprotected subjects (titre ≥ 8) against poliovirus types 1, 2 and 3 at visit 1 (before administration of the additional IPV-Al dose), the geometric mean titres (GMTs) of all three poliovirus types measured before and 28–42 days after administration of the additional IPV-Al dose as well as the booster effect derived from individual serum titre values before and after administration of the additional IPV-Al dose.

Safety endpoints were solicited systemic AEs and ISRs. Solicited AEs included redness, swelling, pain and itching on the injection site of IPV-Al and concomitant vaccines, and general adverse events such as pyrexia, irritability, tiredness, hypersomnia, decreased appetite and vomiting.

### 2.5 Statistical analyses

Results from statistical analyses were presented with estimates and 95% confidence intervals (CI). For log-transformed analyses, the anti-log transformation was applied before the presentation. The software SAS® version 9.4 was used for calculating the results of this trial. There was no interim analysis.

#### 2.5.1 Sample size calculation

For sample size calculation, the observed post-booster seroprotection rate for poliovirus type 1 in the previous booster trial (96.9%) was used. The exact 95% CI was then derived, depending on a hypothetical range of possible sample sizes. With a sample of 150 subjects, the lower limit of the 95% CI would be 89%, which was judged as the minimum acceptable number. This minimum sample size was met since 163 subjects were enrolled and received IPV-Al in this trial.

#### 2.5.2 Immunogenicity and safety analyses

Numerical data were presented in summary tables by the number of subjects, Arithmetic mean and standard deviation and/or geometric mean and coefficient of variation (CV), where applicable, were used to present continuous data. Categorical data were presented by frequency and percentage of subjects as well as number of events, where applicable.

The analysis of the primary and secondary endpoints was based on the per protocol (PP) population, defined as all subjects who completed the trial without any major protocol deviations. GMTs and median antibody titres were summarised by visit, and the booster effect, the ratio of Visit 2 vs Visit 1 titres, derived from individual antibody titre values measured before and 28–42 days after administration of the vaccine, was calculated for each subject and summarised using GMT ratio (GMTR), CV, range and 95% CI. All safety parameters were evaluated for the safety analysis set (SAF), which included all enrolled subjects who received the IPV-Al vaccination at visit 1.

## 3 Results

### 3.1 Population characteristics

**Fig. 1:**
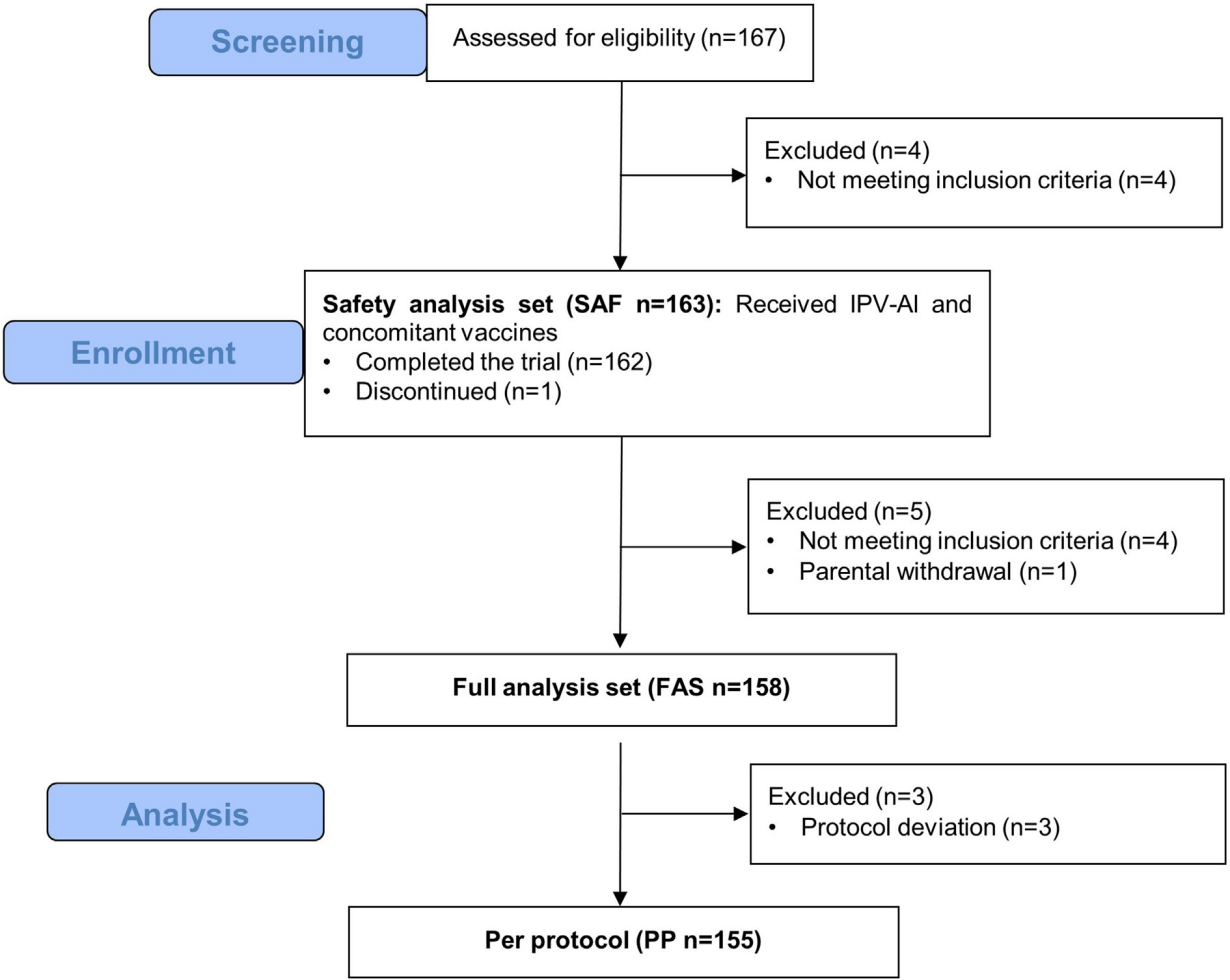
**Flowchart of patients enrolled in the trial.** 167 subjects were screened for eligibility. 163 were enrolled and received the trial vaccination (safety analysis set, SAF). Five subjects were excluded from the full analysis set (FAS), and three subjects were excluded from the per protocol analysis (PP) due to protocol deviation. 162 subjects completed the trial and 1 discontinued. The analysis sets were 163 subjects for SAF, 158 for FAS, and 155 for PP.

The trial was conducted between 20 November 2020 (first subject visit 1) and 03 May 2021 (last subject visit 2). A total of 167 subjects were screened for eligibility, corresponding to 48% of the eligible candidates from the primary and booster trials. 163 were enrolled and received the trial vaccination. The analysis sets were 163 subjects for SAF, 158 for full analysis set (FAS,) and 155 for PP, as illustrated in the CONSORT flowchart in Fig. 1. Demographic and baseline characteristics of the sample are presented in Table 1.

**Table 1:** Demography and baseline characteristics.

		**Total**
Safety population		163

Sex, n (%)	Female	86 (52.8)
	Male	77 (47.2)
Age (days)	Mean (SD)	1460 (22.3)
	Min; Max	1432; 1560

Race, n (%)	American Indian or Alaska Native	157 (96.3)
	Black or African American	3 (1.8)
	Unknown	3 (1.8)

Height (cm)	Mean (SD)	100.7 (4.0)
	Min; Max	91.0; 110.5

Weight (kg)	Mean (SD)	16.5 (2.7)
	Min; Max	12.5; 27.1

(%) = Percentage of subjects in sub-category. SD = Standard deviation.

Subjects received IPV-Al during primary, booster and additional vaccination, and had their blood collected at 4 years of age during visit 1 (pre-vaccination) and visit 2 (post-vaccination). Analysis set: safety analysis set.

All 163 subjects received concomitant vaccines. Before the current trial, 149 (91.4%) subjects received DTP-Hib, 141 (86.5%) subjects received Hepatitis A vaccine and 111 (68.1%) subjects were immunised against influenza. At visit 1, 154 (94.5%) subjects received DTwP/TdaP, 160 (98.2%) received Varicella zoster vaccine and seven (4.3%) subjects received DTP-Hib. At visit 2, two (1.2%) subjects received influenza vaccine.

### 3.2 Immunogenicity

At visit 1, 138 (89%) subjects were seroprotected against poliovirus type 1, 155 (100%) subjects against poliovirus type 2 and 141 (91%) subjects against poliovirus type 3 (Table 2). 28–42 days after the trial vaccination, 154 (99.4%) subjects were seroprotected against poliovirus type 1, and all subjects (100%) were seroprotected against poliovirus type 2 and 3 (Table 2).

**Table 2:** Seroprotection rate and geometric mean titres at visit 1 and 2, by poliovirus type.

	Visit 1	Visit 2
Poliovirus type 1	N = 155	N = 155
Seroprotection	138 (89%)	154 (99.4%)
GMT (CV%)	222.9 (305.2)	5858 (255.9)
Median	256	8192
95% CI	154; 323	4250; 8073
Min; Max	1.4; 32,768	2.0; 185,364

Poliovirus type 2	N = 155	N = 155
Seroprotection	155 (100%)	155 (100%)
GMT (CV%)	829.9 (169.6)	11,534 (141.1)
Median	724	11,585
95% CI	669; 1029	9693; 13,724
Min; Max	11.3; 46,341	256.0; 370,728

Poliovirus type 3	N = 155	N = 155
Seroprotection	141 (91%)	155 (100%)
GMT (CV%)	210.7 (278.3)	6507 (186.8)
Median	362	8192
95% CI	149; 297	5127; 8258
Min; Max	1.4; 11,585	90.5; 185,364

n = number of subjects with titre. GMT = Geometric Mean Titre, defined as EXP(mean(LOG(titre))). CV = 100*sqrt(exp(STD)-1) with standard deviation (STD) based on log-transformed values. CI = confidence interval for GMT. Subjects received IPV-Al during primary, booster and additional vaccination, and had their blood collected at 4 years of age during visit 1 (pre-vaccination) and visit 2 (post-vaccination). Analysis set: per protocol.

The post-vaccination GMTs against poliovirus types 1, 2 and 3 demonstrated a robust increase in antibody titres when compared to pre-vaccination levels. For poliovirus type 1, the subjects had a GMT of 5858 (95% CI: 4250; 8073) at visit 2 compared to 222.9 (154; 323) at visit 1. For poliovirus type 2, GMT was 11534 (9693; 13724) at visit 2 compared to 829.9 (669; 1029) at visit 1. For poliovirus type 3, GMT was 6507 (5127; 8258) at visit 2 and 210.7 (149; 297) at visit 1, as shown in Table 2.

**Fig. 2:**
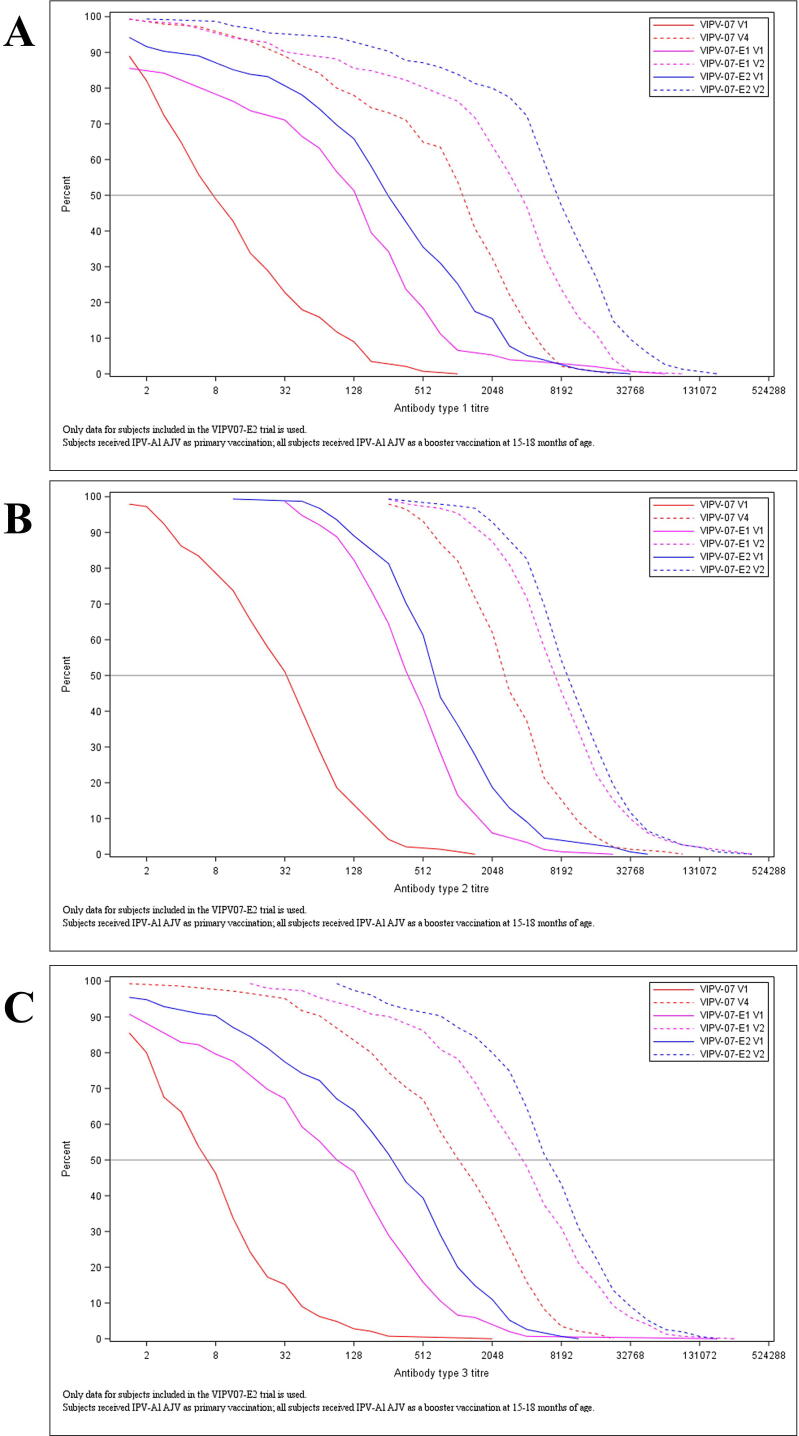
**Reverse cumulative titre distribution for type 1 (A), 2 (B) and 3 (C) antibody titres following primary, booster and additional vaccination with IPV-Al (PP).** Data from primary (VIPV-07) and booster (VIPV-07-E1) trials illustrate only subjects who participated in the present extension. Subjects received IPV-Al as primary, booster and additional vaccination, and had their blood collected at 4 years of age during visit 1 (pre-vaccination) and visit 2 (post-vaccination) of the present trial (VIPV-07-E2). Each figure includes six curves, a pre-booster (V1) solid line and a post-booster vaccination (V2) dotted line for each group. Analysis set: per protocol.

As for the booster effect 28–42 days after the additional dose of IPV-Al, the subjects presented antibody titres 26.3 times higher for poliovirus type 1 (95% CI: 20.10; 34.38), 13.9 times higher for type 2 (10.91; 17.70) and 30.9 times higher for type 3 (22.94; 41.56) at visit 2 compared to visit 1 (Table 3). The booster effect is also illustrated in the reverse cumulative titre distribution curves in Fig. 2.

**Table 3:** Analysis of the boosting factor.

**Poliovirus type**	**n**	**GMTR(CV%)**	**95% CI**	**Min; Max**
Polio type 1	155	26.3(210.5)	(20.10,34.38)	0.5; 2048.0
Polio type 2	155	13.9(189.6)	(10.91,17.70)	1.0; 1448.2
Polio type 3	155	30.9(234.6)	(22.94,41.56)	0.7; 4096.0

n = number of subjects with titre. GMTR = Geometric Mean booster factor, defined as EXP(mean(LOG(booster factor))). CV% = 100*sqrt(exp(STD)-1) with STD based on log-transformed values. CI = confidence interval for GMTR. Subjects received IPV-Al during primary, booster and additional vaccination, and had their blood collected at 4 years of age during visit 1 (pre-vaccination) and visit 2 (post-vaccination). Analysis set: per protocol.

### 3.3 Safety evaluation

No serious AEs were reported. A total of 150 (92%) subjects reported 678 AEs, of which 259 were systemic AEs (111 subjects) and 419 ISRs (146 subjects). The vast majority (90.2%) of the subjects experienced at least one mild AE; 33.1% had at least one moderate AE and 1.2% had at least one severe AE (Table 4). A total of 71.8% of the subjects reported at least one AE that was considered certainly or possibly related to vaccination by the investigator.

**Table 4:** Summary of adverse events and temperature reactions following additional vaccination with IPV-Al.

	**Total** **n (%) E**
**Any AEs**	**150 (92%) 678**
Mild	147 (90.2) 578
Moderate	54 (33.1) 97
Severe	2 (1.2) 2
**Temperature reaction**	
>= 37.5 °C	64 (39.3) 64
>= 38.5 °C	23 (14.1) 23

n (%) = number (percentage) of subjects with AE; E = number of AEs (adverse events). Analysis set: safety analysis set (n = 163).

The most common systemic AEs were pyrexia, fatigue, irritability, decreased appetite, hypersomnia, and somnolence (Table 5). Overall, 64 (39.3%) subjects experienced temperature ≥ 37.5 °C, and 23 (14.1%) subjects had temperatures ≥ 38.5 °C (Table 4). The highest reported temperature was 40.8 °C.

**Table 5:** Summary of the most commonly reported solicited systemic adverse events following additional vaccination with IPV-Al.

**Preferred term**	**System organ class**	**Total** **n (%) E**
**Any systemic AEs**		**111 (68.1) 259**
Pyrexia	General disorders and adm	65 (39.9) 65
Fatigue	General disorders and adm	24 (14.7) 24
Irritability	Psychiatric disorders	62 (38.0) 62
Decreased appetite	Metabolism and nutrition disorders	44 (27.0) 44
Hypersomnia	Nervous system disorders	22 (13.5) 23
Somnolence	Nervous system disorders	19 (11.7) 19

Adm = administration disorders; n (%) = number (percentage) of subjects with AE; E = number of AEs (adverse events). Analysis set: safety analysis set (n = 163).

In total, 35.6% of the subjects had ISRs related to IPV-Al (right deltoid muscle), 32.1% had ISRs related to the Varicella vaccine (left deltoid muscle), whereas 89.6% had ISRs related to the DTwP/TdaP vaccination and 71.4% related to DTP-Hib vaccination (left anterolateral thigh) (Table6). The reported ISRs were either injection site redness or swelling, and of either mild or moderate intensity.

**Table 6:** Related injection site reactions following booster vaccination with IPV-Al.

	**Varicella** **n (%) E**	**DTwP/TdaP** **n (%) E**	**DTP-Hib** **n (%) E**	**IPV-Al** **n (%) E**
Number of subjects vaccinated	159	154	7	163
**Any ISR**	51 (32.1) 81	138 (89.6) 225	5 (71.4) 14	58 (35.6) 92
**Most common ISR by preferred term**				
Redness	20 (12.6) 20	40 (26.0) 40	4 (57.1) 4	21 (12.9) 21
Swelling	22 (13.8) 22	44 (28.6) 45	5 (71.4) 5	22 (13.5) 23
**By intensity**				
Mild Treatment Emergent ISR	51 (32.1) 79	117 (76.0) 187	5 (71.4) 14	58 (35.6) 90
Moderate Treatment Emergent ISR	1 (0.6) 2	33 (21.4) 38	0	1 (0.6) 2

N (%) = number (percentage) of subjects with ISR; E = number of injection reactions ISRs. Analysis set: safety analysis set (n = 163).

Overall, most of the reported AEs had early onset and were transient, beginning within two days after the vaccination and lasting less than two days.

## 4 Discussion

The results from this phase 4 extension trial showed that IPV-Al induces a persistent immune memory in healthy subjects who had received three primary vaccinations with this vaccine at 2, 4, and 6 months of age plus one booster dose at 15–18 months of age. The antibody titres persisted at 4 years of age, i.e., 2.5 years after the previous dose, and were above the seroprotection threshold for 89% of the subjects with regards to poliovirus type 1, 100% for type 2 and 91% for type 3.

At study entry, 17 subjects had antibody titres below the protective level for poliovirus type 1, and 14 subjects for poliovirus type 3. Three subjects were below the protective threshold for both type 1 and type 3 poliovirus. 28–42 days after the additional dose given at age 4, a strong booster effect was observed resulting in all but one subject to be seroprotected at the end of the trial. The one subject that remained below the seroprotection threshold for poliovirus type 1 was known to also respond poorly to the vaccinations in the previous trials. The poor response was specific to poliovirus type 1 and is likely due to an individual-specific characteristic since the antibody titres for poliovirus types 2 and 3 were found to be above the protective threshold in that subject.

In the present trial, the pre-vaccination GMTs were lower than the GMTs after the previous IPV-Al vaccination (fourth dose, booster) at the age of 15–18 months, indicating the natural antibody decay over time. An additional dose of IPV-Al was given to evaluate the presence of immune memory after the primary and booster vaccination. The vaccinees experienced a pronounced anamnestic response induced by the additional dose administered 2.5 years after the previous booster dose [7]. In addition, GMTs after the additional dose were higher than that following the primary and booster vaccination series for all three poliovirus types (Fig. 2).

No fatalities or serious AE were reported during the trial and 99.4% of the participants completed the trial. Of the 259 reported systemic AEs, 90.2% were considered mild, with pyrexia, irritability and decreased appetite being the most common. The ISRs were mainly redness and swelling at the injection site and were considered of mild intensity and short duration. IPV-Al did not seem to alter the safety profile of the concomitant vaccines as the reported ISRs were known and expected. Therefore, the vaccine was considered safe when administered with different concomitant vaccines.

Since vaccines containing aluminium pose a greater risk of eliciting injection site granulomas [14], in particular when more than one aluminium containing vaccine is concomitantly administered, the presence of granulomas, itching nodules, or skin changes at the previous injection sites of IPV-Al were also investigated. However, no subjects reported any of the known aluminium related ISRs. These results, combined with the data from the previous trials, do not indicate a specific safety concern regarding the presence of Al(OH)_3_ in the IPV composition. Overall, the IPV-Al was well tolerated with mild and transient AEs when administered as an additional dose to children who were previously vaccinated with three primary doses and a booster dose.

Not all subjects who were booster vaccinated with IPV-Al AJV during the previous VIPV-07 E1 trial could be reached to participate in this trial. Other reasons such as exclusion criteria (previous OPV administration), COVID pandemic and the vaccination calendar in Panama prevented the recruitment of more subjects. As a result, 48% of the eligible candidates from the primary and booster trials were screened. However, no differences in the antibody response induced by the booster dose in the previous trial were observed in the subset of subjects participating in the present trial in comparison with the subjects not recruited (data not shown). This indicates that the current study population is representative of the whole eligible group.

## 5 Conclusions

The results of the present trial demonstrated that based on the individual antibody titres and seroprotection rates, the vaccination with an adjuvanted reduced dose of IPV induced a persistent immune memory. The observed robust anamnestic response is likely to protect the subjects when exposed to polio later in life. The additional vaccination with IPV-Al at 4 years of age, i.e., 2.5 years after the previous dose, resulted in antibody levels above the seroprotection threshold for poliovirus type 1, 2 and 3 in virtually all subjects. The IPV-Al vaccine was well tolerated, with no serious AEs reported. The IPV-Al was shown to be an immunogenic and safe addition to increase the availability of inactivated polio vaccines that are critical for the success of the current phase of the polio eradication initiative.

## 6 Disclosures

XSL have no conflicts of interest to disclose. CS, LME, MKC, HHK and NT are employees of AJ Vaccines A/S. DBC is a former employee of AJ Vaccines A/S.

## Author contributions

XSL, TP, CO, JSJ, LME and DBC made substantial contributions to the conception and design of the trial as well as to the acquisition and analysis of data. MH and RD were responsible for the enrolment of the participants and following all study procedures, including data acquisition. CS was responsible for the antibody determinations. All authors contributed to the interpretation of the data and were involved in the reviewing and editing of the manuscript. All authors gave final approval and agreed to be accountable for all aspects of the work.

## Funding

This work was supported, in part, by the Bill & Melinda Gates Foundation [OPP1095741]. Under the grant conditions of the Foundation, a Creative Commons Attribution 4.0 Generic License has already been assigned to the Author Accepted Manuscript version that might arise from this submission.

## Declaration of Competing Interest

The authors declare that they have no known competing financial interests or personal relationships that could have appeared to influence the work reported in this paper.
